# Functional role of *FvMdm10* in stress response, pathogenicity, and fumonisins production in *fusarium verticillioides*

**DOI:** 10.1080/21505594.2025.2555419

**Published:** 2025-09-11

**Authors:** Zehua Zhou, Huacui Wang, Jie Liu, Jiahong Yao, Jie Zhang, Rui Hou, Won Bo Shim, Dingfu Xiao, Tuyong Yi

**Affiliations:** a Hunan Provincial Key Laboratory for Biology and Control of Plant Pests, Hunan Agricultural University, Changsha, China; b College of Plant Protection, Hunan Agricultural University, Changsha, China; c Livestock and Poultry Variety Creation Center Beef Cattle Health Breeding Team, Yuelushan Laboratory, Changsha, China; d College of Plant Protection, Nanjing Agricultural University, Nanjing, China; e Department of Plant Pathology and Microbiology, Texas A&M University, College Station, TX, USA; f College of Animal Science and Technology, Hunan Agricultural University, Changsha, China

**Keywords:** *Fusarium verticillioides*, *FvMdm10*, fumonisins, lipid droplets

## Abstract

Endoplasmic reticulum-mitochondrial encounter structure (ERMES), the protein complex that tethers mitochondria and endoplasmic reticulum, is critical for mitochondrial functions and various cellular processes. Our preliminary studies showed that mitochondrial integrity was critical for fumonisin biosynthesis. However, the biological roles of ERMES and its subunits in phytopathogenic fungi, especially their roles in the regulation of fumonisin biosynthesis remain ambiguous. In this current study, we investigated the biological functions of FvMdm10, the core component of ERMES, in the fumonisin-producing fungus *Fusarium verticillioides*. The gene-deletion mutant ΔFvMdm10 exhibited attenuated virulence on intact maize kernels compared with the wild-type strain M3125, which partially attributed to impaired vegetative growth and conidia germination. In addition, ΔFvMdm10 became more resistant to QoIs when compared to M3125. Furthermore, we observed anomalous vacuole morphology in ΔFvMdm10, subsequently leading to decreased FB_1_ production. Noticeably, ∆FvMdm10 takes advantage of accumulated lipid droplets to overcome the oxidative stress generated by H_2_O_2_. Taken together, our results showed that *FvMdm10* is critical for fungal development, stress response, lipid biogenesis and fumonisins biosynthesis in the toxigenic fungus *F. verticillioides*.

## Introduction

The eukaryotic cell is a complex entity that performs distinct functions that are interconnected and interdependent. Organelles are components that fulfill specific biochemical tasks and require vigilant regulations [1]. While the compartmentalization of cells into organelles ensures that incompatible biochemical activities remain separated, communication between adjacent organelles is necessary for the exchange of signal molecules and metabolites [1,2]. These communications rely on membrane contact sites (MCSs) or vesicular trafficking systems [3,4], which provide a faster, more direct and reciprocal movement of fundamental metabolites across different organelles [5,6]. Studies conducted over the past decade have demonstrated the existence of various MCSs, which bring different organelles into close spatial proximity and promote direct inter-organelle communication [4,7]. In contrast, any disruption in these communications will lead to defects in cellular growth and development that can result in degenerative diseases [4,7,8].

Mitochondria, the most important organelle in the cell, are indispensable for a wide range of fundamental cellular activities, including ATP biosynthesis, phospholipid homeostasis, ROS generation, Ca^2+^ signaling and heme biosynthesis [9–11]. Therefore, physical contact between mitochondria and different cellular components, such as endoplasmic reticulum (ER) [12], lipid droplet (LD) [13], vacuole [14] and peroxisome [15], are well established. Significantly, a specialized protein complex designated as ER-mitochondrial encounter structure (ERMES), which tethers mitochondria and ER, has been the focus of intensive research. ERMES is comprised of four subunits which include Mdm10, Mdm34, Mdm12 and Mmm1 [16]. Although minor functional divergences exist among these proteins, these are all essential for ERMES formation across the fungal kingdom [9,16]. To date, ERMES subunits have been characterized in some important fungal species such as *Aspergillus fumigatus*, *Candida albicans*, *Neurospora crassa* and *Saccharomyces cerevisiae* [17–19]. In yeast cells, it has been reported that ERMES regulates ER and mitochondrial functions [20,21]. In addition, evidence showed that ERMES subunits Mdm10 and Mdm12 contribute to the mature peroxisome abundance [8]. However, further studies are required to present a clearer understanding of its roles in other plant pathogenic fungi.

Maize is a major grain commodity in the world and has diverse industrial uses (FAO, 2021). Stalk rot and ear rot diseases caused by the fungal pathogen *Fusarium verticillioides* are significant maize production concerns worldwide [22]. In particular, ear rot not only reduces the yield and quality of maize but also the infestation with mycotoxins fumonisins produced by the fungus, particularly the most prevalent form fumonisin B_1_ (FB_1_), poses a significant risk to human health and food safety [22,23]. When compared to another important group of *Fusarium* mycotoxins trichothecenes such as deoxynivalenol (DON), fumonisins are significantly different in structure, mechanisms of toxicity, biosynthesis pathway and regulatory network [23]. Previous studies showed that mitochondria were associated with DON biosynthesis in *F. graminearum*, and quinone outside inhibitors (QoIs) treatment stimulated DON biosynthesis by altering mitochondrial morphology and dynamics [24]. Recently, ERMES was found to interact with the toxisome via Tri1 protein in *F. graminearum*, and two ERMES subunits were indispensable for the regulation of mitochondrial morphology and DON biosynthesis [21]. Furthermore, our recent publication reported that QoIs treatment drastically reduced the production of fumonisins, demonstrating that mitochondria also participate in fumonisins biosynthesis [25]. However, how mitochondria and ERMES affect fumonisins biosynthesis remains unknown.

In this study, we found that the deletion of *FvMdm10* led to defects in mycelial growth, conidia germination, pathogenicity and FB_1_ production in *F. verticillioides*. In addition, the gene-deletion mutant ΔFvMdm10 exhibited more resistance to mitochondrial respiratory-chain inhibitors when compared to the wild-type progenitor M3125. We also observed lipid droplet accumulation in ΔFvMdm10, which was attributed to enhanced oxidative stress tolerance in the fungal pathogen. Taken together, our findings demonstrated that *FvMdm10* is crucial for fungal development processes, stress responses, lipid droplets biogenesis and fumonisins biosynthesis in *F. verticillioides*.

## Materials and methods

### Fungal strains and culture conditions

*F. verticillioides* wild-type strain M3125 [26] and the derived mutants were grown on potato dextrose agar (PDA) at 28°C in an incubator. Conidia production measurement was conducted following a previous publication [26]. For conidia germination assays, conidia harvested from five-day-old PDA plates were cultivated on water agar (WA) plates and then incubated for 8 h at 28°C in the dark [26]. For fungicide sensitivity assays, all strains were cultured on alkyl ester agar (AEA) medium (6 g of NaNO_3_, 1.5 g of KH_2_PO_4_, 5 g of yeast extract, 0.51 g of MgSO_4_·7 H_2_O, 0.5 g of KCl, 20 mL of glycerin and 16 g of agar powder in 1 L of deionized water) at 28°C. For lipid droplet (LD) staining assays, all strains were incubated in yeast extract peptone dextrose (YEPD) medium (3 g of yeast extract, 20 g of glucose and 10 g of peptone in 1 L of deionized water) at 28°C with agitation (175 rpm) for 36 h. For FB_1_ production assays, all strains were incubated in liquid Myro medium (1 g of NH_4_H_2_PO_4_, 3 g of KH_2_PO_4_, 2 g of MgSO_4_·7 H_2_O, 5 g of NaCl and 40 g of sucrose in 1 L of deionized water, pH 5.9) at 28°C with agitation (175 rpm) for 7 days [26].

### Strain construction

The *FvMdm10* (FVEG_09107) knockout mutant (ΔFvMdm10) was generated based on the homologous recombination strategy (Figure S1) [25]. All unidentified transformants were first screened by PCR assays, and then the null mutants were further verified by qRT-PCR assays. To generate a complementation strain (∆FvMdm10-C), the DNA fragment containing the whole *FvMdm10* gene and the geneticin-resistant gene (*GEN*) was constructed, and subsequently transformed into the protoplasts of the ΔFvMdm10 mutant. All primers used in the current study are listed in Table S1.

### Infection assay of maize kernels

Maize kernel virulence assays were performed following the recent publication [27]. Ten maize kernels were surface sterilized and placed on a glass petri dish with moist filter paper. Conidia of M3125, ∆FvMdm10 and ∆FvMdm10-C were harvested with sterilized water from five-day-old PDA plates, and the conidia suspensions (10 μL of 10^6^ conidia/mL) were inoculated on maize kernels. After incubating at 28°C for 5 days, the infected maize kernels were imaged, then washed with 20 mL sterilized water and the number of conidia was counted with a hemocytometer. The assays were repeated three times with three plates for each experiment.

### Response of ∆FvMdm10 to environmental stresses and fungicides

To evaluate the effects of *FvMdm10* deletion on environmental stress responses, mycelial plugs taken from the edge of a 3-day-old colony were inoculated on PDA plates amended with the following compounds: 20 mM H_2_O_2_, 1 M NaCl, and 0.01% (w/v) SDS, respectively. After incubating for 4 days at 28°C in an incubator, the fungal colony diameter of each plate was measured. The assays were repeated three times with three plates for each experiment.

To determine the sensitivity of M3125, ∆FvMdm10, and ∆FvMdm10-C to QoI fungicides, all strains were inoculated on AEA medium containing a series of concentrations of azoxystrobin and pyraclostrobin, respectively. To suppress the alternative oxidase pathway in *F. verticillioides*, salicylhydroxamic acid (SHAM) was added in a final concentration of 50 μg/mL. After incubating for 4 days at 28°C, the fungal colony diameter was measured and the EC_50_ values were calculated [28]. The assays were repeated three times with three plates for each experiment.

### Assays for FvMdm10 deletion on ATP biosynthesis and ergosterol production

For ATP production analysis, M3125, ∆FvMdm10, and ∆FvMdm10-C were cultured in Myro medium at 28°C with agitation (175 rpm) for 2 days. Mycelia were harvested and ground with liquid nitrogen. The quantification of ATP concentration was conducted with the ATP Content Assay Kit (Solarbio). For ergosterol production assays, all strains were cultured in YEPD at 28°C with agitation (175 rpm) for 2 days, then mycelia were harvested and ground with liquid nitrogen, weighed at 0.2 g and dissolved with 4 mL ethyl alcohol, and then left overnight at room temperature. The ergosterol content of each strain was measured as previously described [21]. The assays were repeated three times with three replicates for each experiment.

### Assays for fumonisins biosynthetic gene expression and FB_1_ production

To determine the transcription levels of fumonisins biosynthetic genes, all strains were cultured in Myro medium at 28°C for 2 days, and mycelia were collected for RNA extraction and cDNA synthesis. The qPCR assays were performed with a qPCR Master Mix kit (Vazyme, Q411). The relative transcription level of target genes with the GAPDH gene as an endogenous standard was calculated using the 2^–∆∆CT^ method [29]. The experiments were repeated three times.

For FB_1_ production analysis, M3125, ∆FvMdm10, and ∆FvMdm10-C were cultured in Myro medium at 28°C with agitation (175 rpm) for 7 days in the dark followed previous publication [25,26]. Subsequently, mycelia along with 2 mL culture broth of all strains were collected, respectively. FB_1_ production was analyzed using an ELISA detection kit (Wise) following the manufacturer’s protocols. The experiments were repeated three times.

### Fluorescence microscopy and image processing

CMAC (KeyGen) was used for vacuole staining as described previously [26]. The vacuolar structure was examined under a fluorescence microscope PA53FS6 (Motic) with a UV filter. Nile Red (Solarbio) was used for lipid droplets (LDs) staining as described previously [30]. Briefly, M3125 and ∆FvMdm10 were cultured in YEPD medium at 28°C with agitation (175 rpm) for 36 h, then hyphae or conidia of each strain were incubated in Nile Red staining solution (2.5 mg/mL Nile Red Oxazone and 0.02 g/mL polyvinylpyrrolidone) in 50 mM Tris-maleate buffer (pH 7.5) for 60 seconds, then LDs in hyphae or conidia were examined under a fluorescence microscope with a RFP filter. For quantification of the number of LDs, three counts of 100 conidia were recorded. Means (± SD) presented the average number of LDs per conidium calculated in three independent experiments.

### Transmission electron microscopy (TEM) assays

For TEM examination, M3125, ∆FvMdm10 and ∆FvMdm10-C were cultured in a YEPD medium at 28°C with agitation (175 rpm) for 36 h. Mycelia of all strains were collected and washed with sterilized water, then fixed in 2.5% glutaraldehyde at room temperature for 2 h in the dark and kept at 4°C. The subsequent sample preparation and testing were performed by Pinuofei Biological Technology Co., Ltd (Wuhan, China).

## Results

### FvMdm10 is crucial for fungal development and pathogenicity

**Figure 1.**
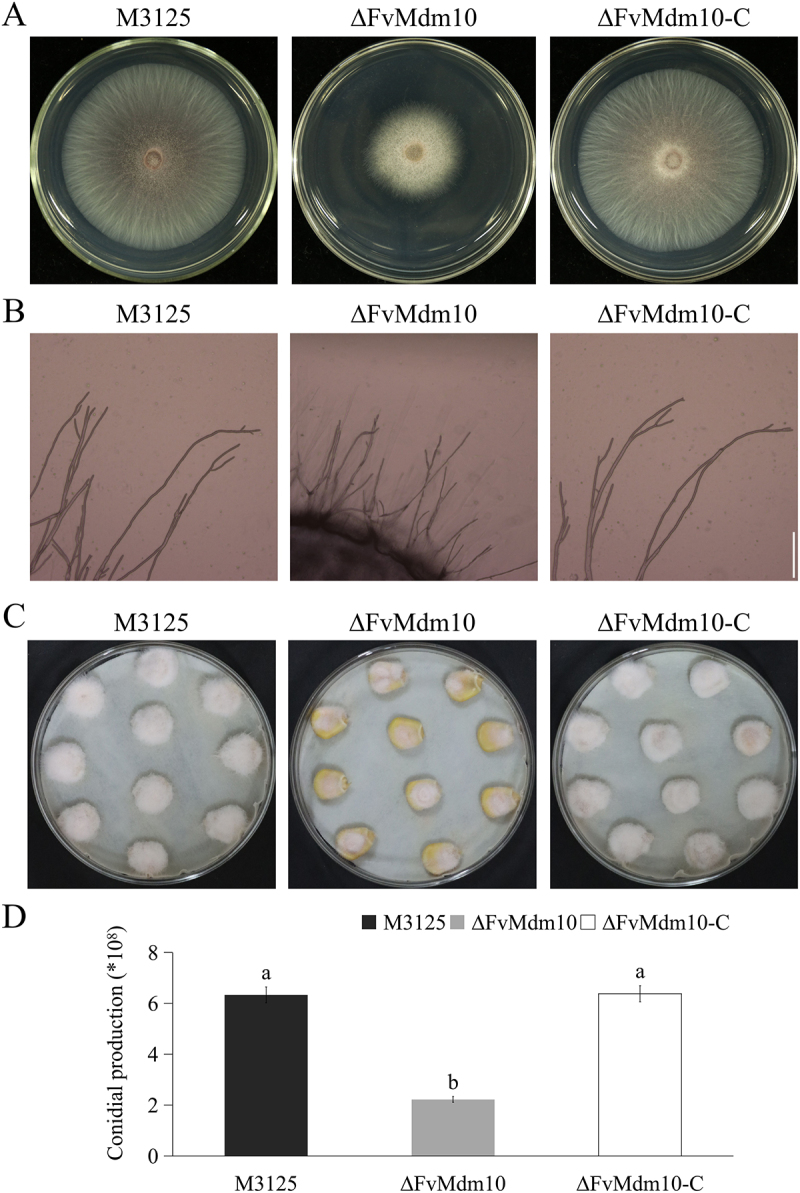
*FvMdm10* is indispensable for vegetative growth and pathogenicity in *F. verticillioides*. (a) Colony morphology of the wild-type M3125, ΔFvMdm10 and complementary mutant ΔFvMdm10-C on PDA plates at 28°C for 4 days. (b) Hyphal morphology of M3125, ΔFvMdm10 and ΔFvMdm10-C on PDA plates. Bar = 100 μm. (c) Pathogenicity of M3125, ΔFvMdm10 and ΔFvMdm10-C on the unwounded maize kernel. (d) The quantity of conidia of each strain grown on maize kernels was analyzed via a hemocytometer under a light microscope. Values on the bars followed by the same letter are not significantly different (*p* < 0.05) based on Fisher’s LSD test.

As indicated, ∆FvMdm10 was significantly reduced in vegetative growth rate when compared to M3125 and ∆FvMdm10-C, with the colony diameter of 2.7, 5.1, and 5.0 cm, respectively (Figure 1(a)). Notably, no difference in the hyphal morphology was observed between M3125 and ∆FvMdm10 (Figure 1(b)). The pathogenicity assay on maize kernels showed that ∆FvMdm10 exhibits decreased virulence and less conidia production on maize kernels when compared to M3125 (Figure 1(c,d)). Our results suggested that *FvMdm10* plays a vital role in vegetative growth and pathogenicity in *F. verticillioides*.

### FvMdm10 is involved in the regulation of asexual reproduction

**Figure 2.**
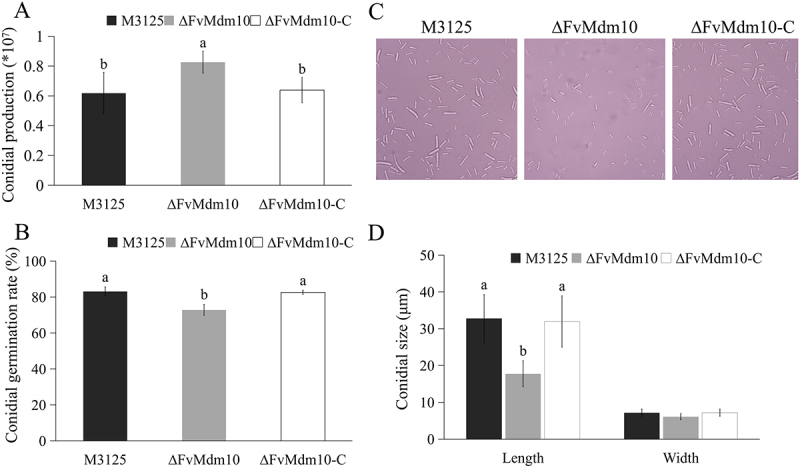
Involvement of *FvMdm10* in regulating conidiogenesis in *F. verticillioides*. (a) Conidial production of M3125, ΔFvMdm10 and ΔFvMdm10-C. Conidia were harvested from the strains cultured on 7-cm PDA plates at 28°C for 4 days. (b) Conidial germination rate of M3125, ΔFvMdm10 and ΔFvMdm10-C. Conidial germination rate of each strain (*n* = 100) was examined after incubating at 28°C for 8 h in the dark on WA plates. (c) Deletion of *FvMdm10* resulted in conidial morphological defects. (d) The average conidia length and width of each strain were measured with 50 conidia. Values on the bars followed by the same letter are not significantly different (*p* < 0.05) based on Fisher’s LSD test.

In the current study, we found an increase in conidia production in ∆FvMdm10 when cultured on PDA medium in comparison with M3125 (Figure 2(a)). However, the conidia germination rate of ∆FvMdm10 was decreased when compared with M3125 and ∆FvMdm10-C (Figure 2(b)). When examined under the microscope, we found that the average length of M3125 conidia was 32.8 ± 6.5 µm, while ∆FvMdm10 conidia showed only 17.8 ± 3.5 µm (Figure 2(c,d)). Collectively, our results showed the regulatory roles of *FvMdm10* in conidia production and germination in *F. verticillioides*.

### FvMdm10 was critical for stress tolerance

**Figure 3.**
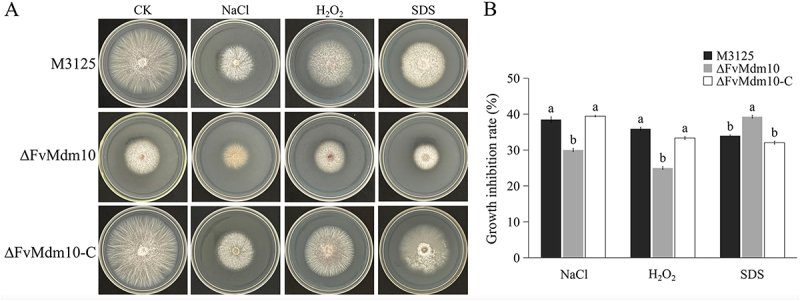
Sensitivity of the ΔFvMdm10 mutant to different stress factors. (a) The colony morphology of M3125, ΔFvMdm10 and ΔFvMdm10-C on PDA medium containing 20 mM H_2_O_2_, 1 M NaCl, and 0.01% SDS. The colony diameter was measured after 4 days of incubation at 28°C. (b) The relative growth inhibition rates of all strains under different stress factors (normalized to colony diameter on PDA plates). Values on the bars followed by the same letter are not significantly different (*p* < 0.05) based on Fisher’s LSD test.

To determine the role of *FvMdm10* in responses to various stress agents, we measured the growth rate of M3125, ∆FvMdm10 and ∆FvMdm10-C on 1 M NaCl (osmotic stressor), 0.01% w/v SDS (cell wall-damaging agent) and 20 mM H_2_O_2_ (oxidative stressor). Our results showed that ∆FvMdm10 exhibited increased sensitivity to SDS but decreased sensitivity to H_2_O_2_ and NaCl when compared with M3125 and ∆FvMdm10-C (Figure 3), suggesting the pivotal role of *FvMdm10* in stress response in *F. verticillioides*.

### Disruption of FvMdm10 significantly increases lipid droplet accumulation

**Figure 4.**
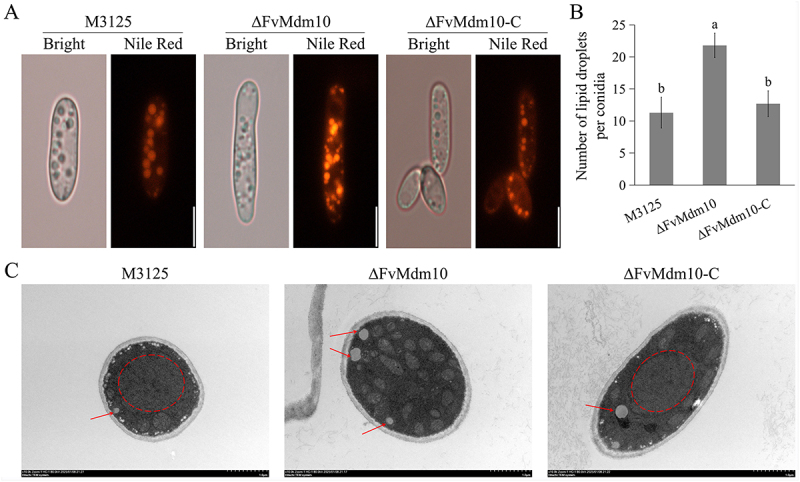
The *FvMdm10* is involved in lipid droplet (LD) biogenesis. (a) LDs accumulation in the conidia of ΔFvMdm10 as compared with M3125 and ΔFvMdm10-C. The conidia of all strains were stained with Nile red to examine LD accumulation. Bar = 20 μm. (b) The average number of LDs per conidia is shown for the M3125 and ΔFvMdm10 cultured in YEPD media, which was counted with 50 conidia. (c) Ultrastructural morphology of the hyphae of M3125, ΔFvMdm10 and ΔFvMdm10-C was examined by TEM. The typical LDs are labeled with red arrows. The round and large vacuoles are indicated with red dashed boxes. Scale bars are indicated in the images. Values on the bars followed by the same letter are not significantly different (*p* < 0.05) based on Fisher’s LSD test.

As results showed, ∆FvMdm10 exhibited decreased sensitivity to H_2_O_2_ when compared to M3125. In previous studies, LD biogenesis was linked to resistance against oxidative stress [30,31]. We hypothesized that the increased tolerance to oxidative stress generated by H_2_O_2_ is associated with LD accumulation in ∆FvMdm10. Therefore, qRT-PCR assays were first performed to analyze the transcription levels of key genes associated with lipid metabolism in M3125, ∆FvMdm10 and ∆FvMdm10-C. As indicated, the transcription of selected genes was significantly altered in the absence of *FvMdm10*, with three down-regulated and 3 up-regulated genes (Figure S2a), suggesting that lipid homeostasis was affected in ∆FvMdm10. Subsequently, we examined LD abundance in ∆FvMdm10 with LD dye Nile Red, and the results showed that strong fluorescent signals were observed in both the mycelia and conidia of ∆FvMdm10 compared to M3125 (Figure 4(a), Figure S2b). The quantitative distribution of LDs demonstrated that deletion of *FvMdm10* led to a two-fold increase in LD numbers per conidium grown in the YEPD medium (Figure 4(b)). Accumulated LDs in the hyphae of ∆FvMdm10 were also verified by transmission electron microscopy (TEM) examination (Figure 4(c)). Collectively, these data suggested that *FvMdm10* plays a crucial role in lipid accumulation, and ∆FvMdm10 takes advantage of LD to overcome oxidative stress.

### FvMdm10 was involved in regulating ergosterol but not ATP biosynthesis

**Figure 5.**
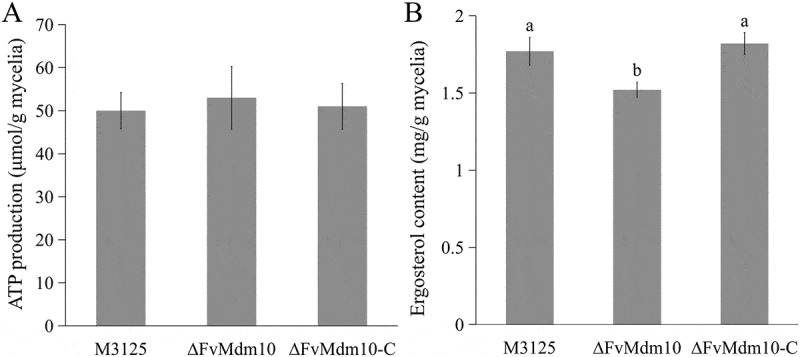
*FvMdm10* deletion affects ergosterol biosynthesis but not ATP production in *F. verticillioides*. (a) Ergosterol content in M3125, ΔFvMdm10 and ΔFvMdm10-C. (b) ATP content in M3125, ΔFvMdm10 and ΔFvMdm10-C. Values on the bars followed by the same letter are not significantly different (*p* < 0.05) based on Fisher’s LSD test.

In the current study, no significant difference was observed in mitochondrial morphology (data not shown) as well as the ATP content between ∆FvMdm10 and its progenitor M3125 (Figure 5(a)), which is not consistent with recent publications that ERMES regulates ATP production [21,32]. However, a significant decrease in ergosterol levels was observed in ∆FvMdm10 (1.52 mg/g mycelia) compared to M3125 and ∆FvMdm10-C (1.77 and 1.81 mg/g mycelia, respectively) (Figure 5(b)). This suggested *FvMdm10* plays a regulatory role in *F. verticillioides* ergosterol metabolism.

### ∆FvMdm10 exhibited low sensitivity to mitochondrial respiratory chain inhibitors

**Figure 6.**
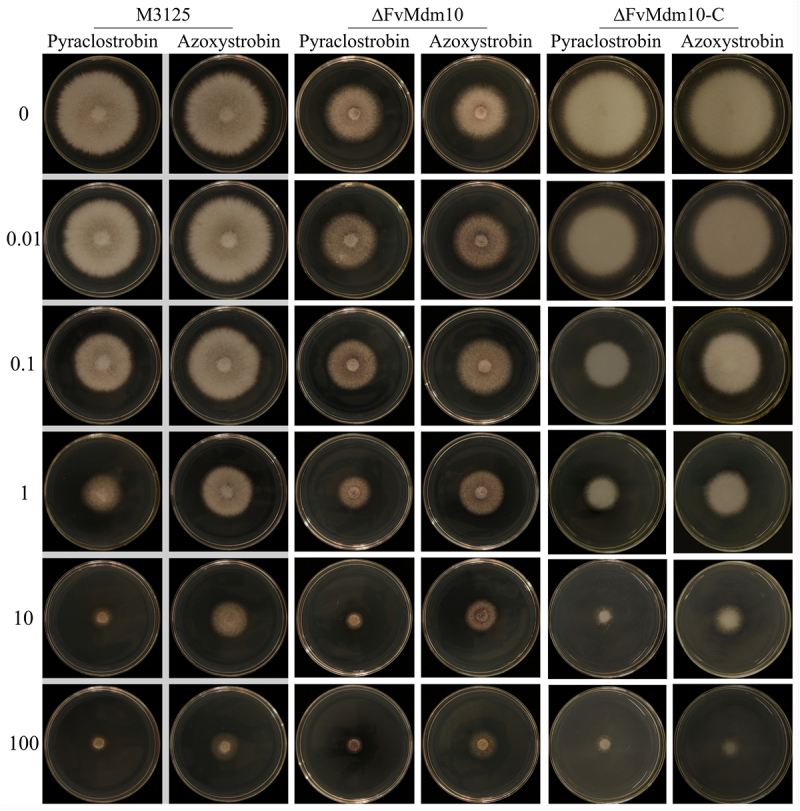
*FvMdm10* mutation significantly increases the resistance to QoI fungicides in *F. verticillioides*. M3125, ΔFvMdm10 and ΔFvMdm10-C were grown at 28°C for 4 days on AEA plates amended with 0.01, 0.1, 1, 10, and 100 μg/mL of azoxystrobin and pyraclostrobin, respectively.

Quinone outside inhibitors (QoIs) and succinate dehydrogenase inhibitors (SDHIs) were used to determine the sensitivity of M3125, ∆FvMdm10 and ∆FvMdm10-C. As indicated, the sensitivity of ∆FvMdm10 toward azoxystrobin and pyraclostrobin was significantly lower than that of M3125 and ∆FvMdm10-C, with the EC_50_ values of 1.99 and 25.31 μg/mL in ∆FvMdm10 as compared with 0.54 and 3.74 μg/mL in M3125, respectively (Table 1, Figure 6). In addition, we found that the expression level of *FvVps13*, the gene associated with the tolerance of mitochondrial respiratory-chain inhibitor, was upregulated in ∆FvMdm10 when compared to M3125 (Figure S3). However, the inhibition rate of M3125 and ∆FvMdm10 showed no significant difference in the presence of 10 μg/mL fluopyram and boscalid, suggesting that *F. verticillioides* strains were insensitive to SDHIs (data not shown). Taken together, these data demonstrated that *FvMdm10* contributes to QoIs sensitivity.

**Table 1.** Sensitivity of M3125, ΔFvMdm10 and ΔFvMdm10-C to QoI fungicides.

Strains	EC_50_ (μg/mL)	
	Pyraclostrobin	Azoxystrobin
M3125	0.5447 ± 0.0739b	3.3421 ± 0.0836b
ΔFvMdm10	1.9918 ± 0.0718a	25.3135 ± 2.3145a
ΔFvMdm10-C	0.6718 ± 0.0831b	2.7430 ± 0.0764c

### FvMdm10 is required for fumonisin biosynthesis and vacuolar morphology

**Figure 7.**
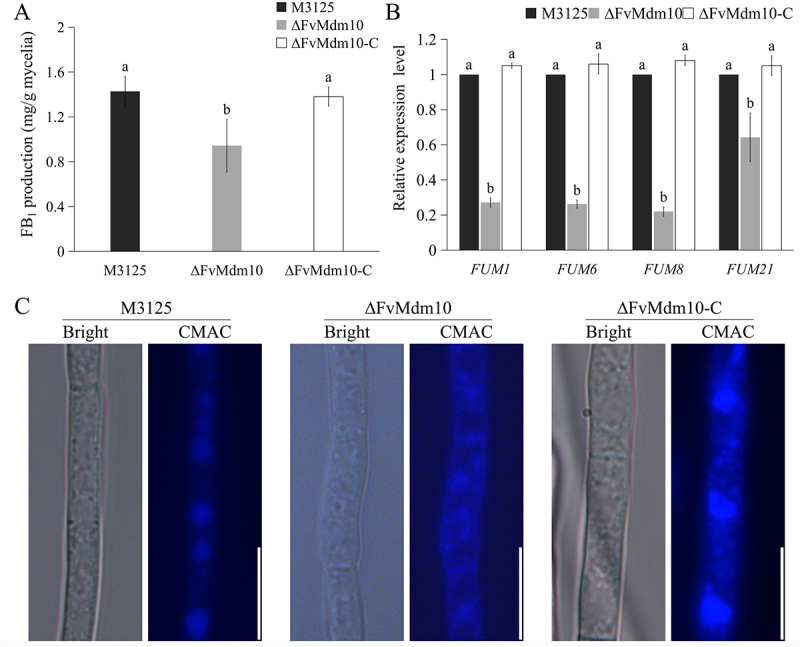
Δ*FvMdm10* is critical for FB_1_ biosynthesis in *F. verticillioides*. (a) FB_1_ production of M3125, ΔFvMdm10 and ΔFvMdm10-C in Myro medium at 28°C for 7 days. (b) The transcription level of *Fum1*, *Fum6*, *Fum8* and *Fum21* was significantly decreased in ΔFvMdm10 when compared with M3125 and ΔFvMdm10-C. (c) Vacuolar structure of the M3125, ΔFvMdm10 and ΔFvMdm10-C revealed by CMAC-staining assays. Bar = 10 μm. Values on the bars followed by the same letter are not significantly different (*p* < 0.05) based on Fisher’s LSD test.

After incubating in Myro medium for 7 days, ∆FvMdm10 produced less FB_1_ when compared with M3125 and ∆FvMdm10-C (Figure 7(a)). By measuring the transcriptional levels of fumonisin biosynthetic genes in ∆FvMdm10 and M3125, we found that the expression levels of *FUM1*, *FUM6*, *FUM8*, and *FUM21* were significantly decreased (Figure 7(b)), demonstrating that *FvMdm10* is critical for fumonisins biosynthesis in *F. verticillioides*.

We also determined the role of *FvMdm10* in maintaining the vacuolar morphology. CMAC staining assays showed that M3125 and ∆FvMdm10-C exhibited large and spherical vacuoles, whereas ∆FvMdm10 exhibited diffused blue fluorescence throughout the fungal hyphae Figure 7(c)). In addition, TEM examinations further showed smaller and irregular vacuoles in ∆FvMdm10 when compared with M3125 and ∆FvMdm10-C (Figure 4(b)). Taken together, our results demonstrated that *FvMdm10* is indispensable for vacuolar morphology and FB_1_ production in *F. verticillioides*.

## Discussion

ERMES, the protein complex connecting mitochondria and ER, is important for the biological functions of these two organelles, but few studies on the specific regulatory roles of ERMES subunits have been reported. Recent publications showed that ERMES deficiency caused by Mdm10 or Mdm12 mutation leads to the dysfunction of the ER, consequently affecting ergosterol biosynthesis [20,21]. Similar results were also obtained in *S. cerevisiae* [33]. In the current study, a significant reduction in ergosterol production was found in ∆FvMdm10, further demonstrating the involvement of ERMES in the regulation of ER functions. Apart from ER-mediated ergosterol biosynthesis, ERMES disruption also influences mitochondrial functions. In *S. cerevisiae*, the Mdm10 mutant exhibited alterations in mitochondrial morphology [34]. Likewise, Mmm34, Mdm12, and Mdm1 mutations also exhibited obvious defects in the mitochondrial structure [18,35,36]. Recently, Song and colleagues found that the ERMES subunit mutants ∆Mdm10 and ∆Mmm1 displayed abnormal mitochondrial morphology, further leading to the decline of ATP production in *F. graminearum* [21]. However, our data showed that Mdm10 deletion did not affect mitochondrial morphology and ATP production in *F. verticillioides*, which can be attributed to species-specific disparities. Intriguingly, we found that ∆FvMdm10 was more resistant to QoIs pyraclostrobin and azoxystrobin when compared to M3125, indicating that the FvMdm10 protein is involved in the response to mitochondrial respiratory-chain inhibitors. Accumulated evidence revealed that the defects in ERMES could be compensated by another MCS designated as a vacuole and mitochondria patch (vCLAMP) [37–39]. Among all vCLAMP components, Mcp1 and Vps13 act as functional effectors, and their overexpression can rescue the respiratory growth defects caused by Mdm10 or Mdm12 mutations [40]. Therefore, we used *S. cerevisiae* Mcp1 and Vps13 as the query and screened predicted proteins in *F. verticillioides*. Although the putative Mcp1 ortholog was not identified, the homolog of *S. cerevisiae* Vps13 was obtained in *F. verticillioides*, and we found that the expression level of *FvVps13* was upregulated in ∆FvMdm10 when compared to M3125. In light of this, we hypothesize that ∆FvMdm10 takes advantage of the upregulation of *FvVps13* to overcome the fungicide stress generated by QoIs. However, detailed molecular research on FvMdm10-mediated response to QoIs is needed.

Fungal mitochondria are crucial to cell growth and intracellular morphogenetic switching [40,41]. Therefore, it is not surprising that any dysfunctions in ERMES integrity will affect mitochondrial morphology and metabolic activity and further lead to growth defects. Consistent with this, mutations of the ERMES subunit exhibited defects in cell wall integrity, which make the mutants more sensitive to cytoderm targeting inhibitors [42,43]. A published report showed that all *S. cerevisiae* ERMES mutants are sensitive to calcofluor white (CFW) as well as caspofungin, and that ΔMdm10 is the most affected [42]. Similar results were also found in *C. albicans* [43]. In the current study, the relative mycelial growth inhibition rate of ∆FvMdm10 is 39.25%, which is higher than that of M3125 (33.99%), indicated that ∆FvMdm10 was more sensitive to the cell wall-damaging agent SDS when compared to M3125, demonstrating that *Mdm10* has a conserved function in regulating cell-wall integrity among filamentous fungi.

Although evidence showed that all ERMES deletion mutants in *S. cerevisiae* did not display a hypersensitivity or resistance to oxidative stress generated by H_2_O_2_ [8], our research found that ∆FvMdm10 was more tolerant to H_2_O_2_ as compared with M3125, with the relative growth inhibition rate of 25.00% and 35.88%, respectively, indicating the species-specific functions of Mdm10 in oxidative stress response. Notably, Liu and colleagues found that LD biogenesis can increase the resistance of fungi to oxidative stress within the host tissue, and reactive oxygen species (ROS) treatment could facilitate the accumulation of LDs in the mycotoxigenic fungus *F. graminearum* [30]. On the contrary, the disruption of LD formation led to the increased sensitivity to ROS such as H_2_O_2_ in *F. graminearum*. Similar outcomes were also obtained by Seo and Shin, who found that mammal cells take advantage of LDs to cope with oxidative stress [32]. Moreover, it is worth mentioning that the interactions between mitochondrial and lipid droplets are important for lipid storage and utilization [44]. In the current study, a higher abundance of LDs in ∆FvMdm10 was observed as compared to M3125 via LD staining assays and TEM examinations, indicating that the accumulated lipid droplets are associated with enhanced oxidative stress tolerance in ∆FvMdm10, further demonstrating that the function of LD in anti-oxidative stress is conserved from mammals to fungi. Although accumulated evidence demonstrated that mitochondrial dynamics were important to lipid storage and utilization [44,45], the connections among FvMdm10, mitochondrial dynamics, and accumulated LDs in *F. verticillioides* remain to be explored.

Conidia are always recognized as the key inoculum in the *Fusarium* plant disease cycle [46,47]. Our research found that ∆FvMdm10 suffered defects in conidiogenesis, including conidia germination and conidial morphology, which further contributed to the attenuated virulence on maize kernels. This outcome demonstrated the necessity of *FvMdm10* in plant infection and pathogenesis. Fumonisins are one of the most important toxic metabolites produced by *Fusarium* species [48], posing a critical threat to human health and causing enormous losses to agricultural production [22,23]. In a recent publication, mitochondria were found to be associated with FB_1_ production [25]. In this study, we found that the mutant ∆FvMdm10 produced less FB_1_ as compared to M3125 (0.94 mg/g mycelia vs 1.43 mg/g mycelia), and the transcription level of key fumonisins biosynthetic genes was significantly suppressed. These data indicated that *FvMdm10* was crucial for the regulation of fumonisins biosynthesis in *F. verticillioides*. Our recent studies found that vacuoles were indispensable for fumonisins biosynthesis [49], and found that mitochondria and vacuoles are functionally linked in *F. verticillioides* [25]. Similarly, the importance of the mitochondria-lysosome connections has been widely reported across organisms [50–52]. In this study, an abnormal vacuolar morphology was found in ∆FvMdm10 when compared with M3125 and ∆FvMdm10-C via microscopic observation and TEM examination, suggesting that ERMES could affect the connections between mitochondria and vacuoles, thereby regulating vacuolar morphology in *F. verticillioides*. However, the molecular mechanism by which FvMdm10 and ERMES regulate fungal vacuoles requires further investigation.

Evidence showed that TOR-pathway mediated LD biogenesis is critical for DON biosynthesis, which could also be affected by rapamycin, the chemical substance that targets the TOR pathway in filamentous fungi, in a dose-dependent manner in *F. graminearum* (unpublished data). Furthermore, LD accumulation was observed under toxin-inducing conditions in *F. graminearum*, which could also be induced by rapamycin [30]. However, our studies found no significant difference in LD accumulation between toxin-non-inducing conditions (YEPD medium) and toxin-induction conditions (Myro medium), nor rapamycin-altered fumonisin biosynthesis in *F. verticillioides* (Figure S4), suggesting that LD biogenesis is not closely associated with fumonisin production, further demonstrating that the regulatory network of fumonisin biosynthesis is distinct when compared with that of DON biosynthesis.

Taken together, our results provide evidence that *FvMdm10* regulates fungal development processes, virulence and stress response. These data suggest that deletion of *FvMdm10* leads to the accumulation of lipid droplets, which ultimately contributes to enhanced oxidative stress tolerance. Moreover, *FvMdm10* deletion resulted in irregular and smaller vacuoles, subsequently leading to decreased FB_1_ production in *F. verticillioides*. Our study will forward understanding of fungal genetics and biology of Mdm10 protein, and provide insights into potential targets to manage *F. verticillioides* contamination on crops.

## Supplementary Material

Figure_S2.tif

Figure_S3.tif

Table S1.doc

Figure_S1.tif

Figure_S4.tif

## Data Availability

The data that support the findings of this study are openly available in ScienceDB at https://doi.org/10.57760/sciencedb.21882, reference number [53].
