# Supplementary figures and images for: Functional role of FvMdm10 in stress response, pathogenicity, and fumonisins production in fusarium verticillioides
Source: Virulence. 2025 Sep 11;16(1):2555419. doi: 10.1080/21505594.2025.2555419 (PMC12427440; doi:10.1080/21505594.2025.2555419)

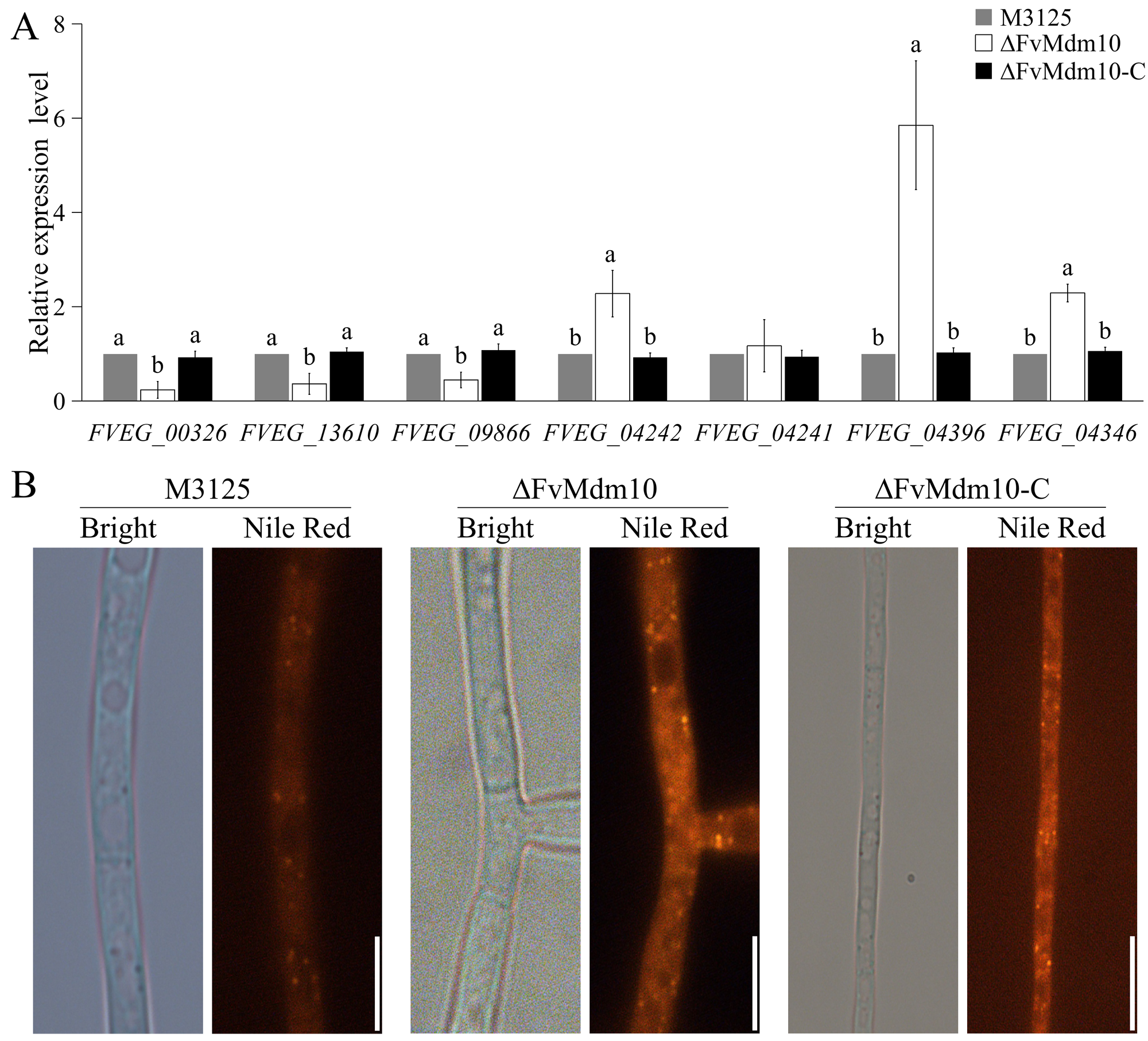

Supplement: Figure_S2.tif [file KVIR_A_2555419_SM8535.tif]

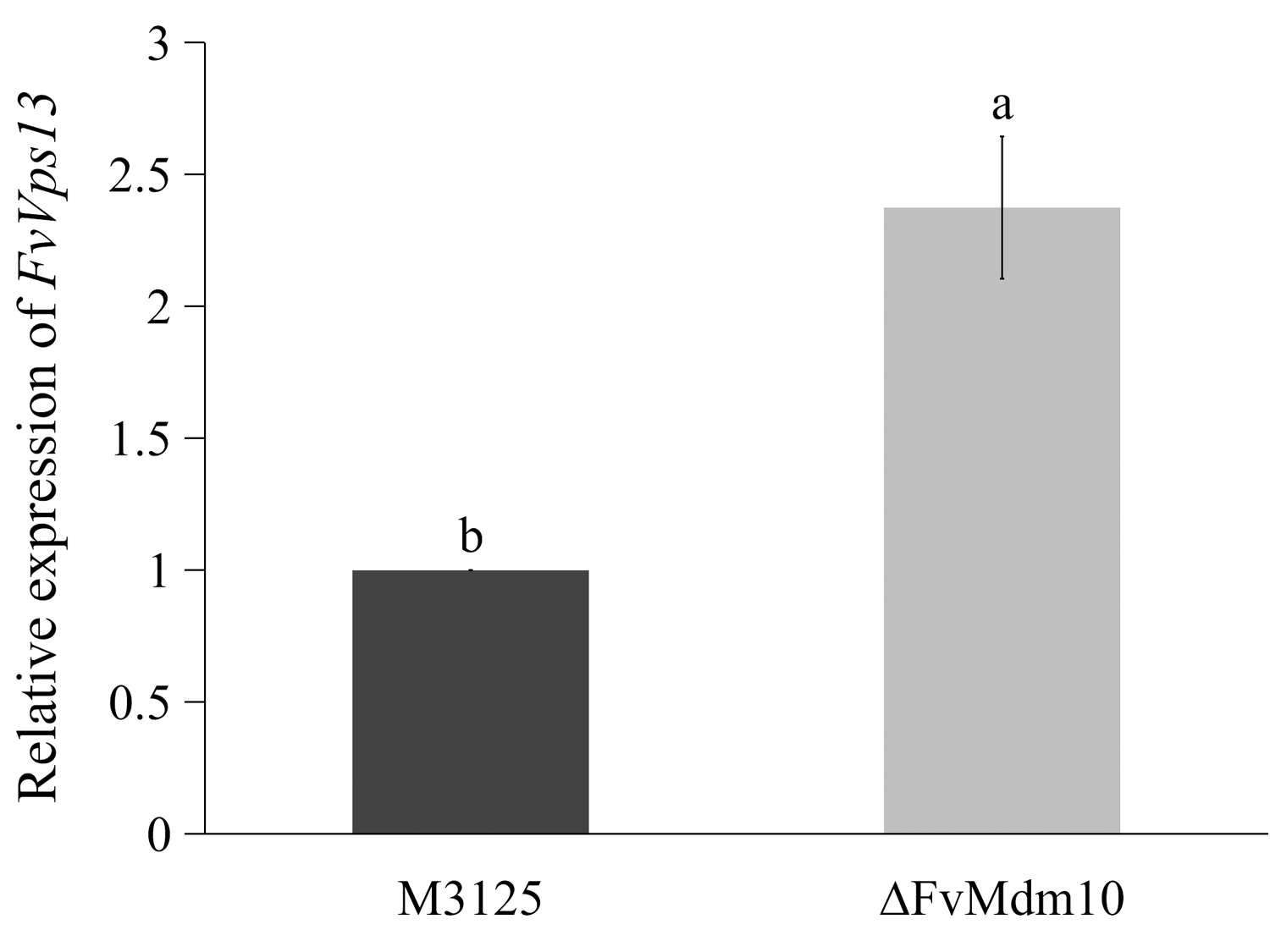

Supplement: Figure_S3.tif [file KVIR_A_2555419_SM8534.tif]

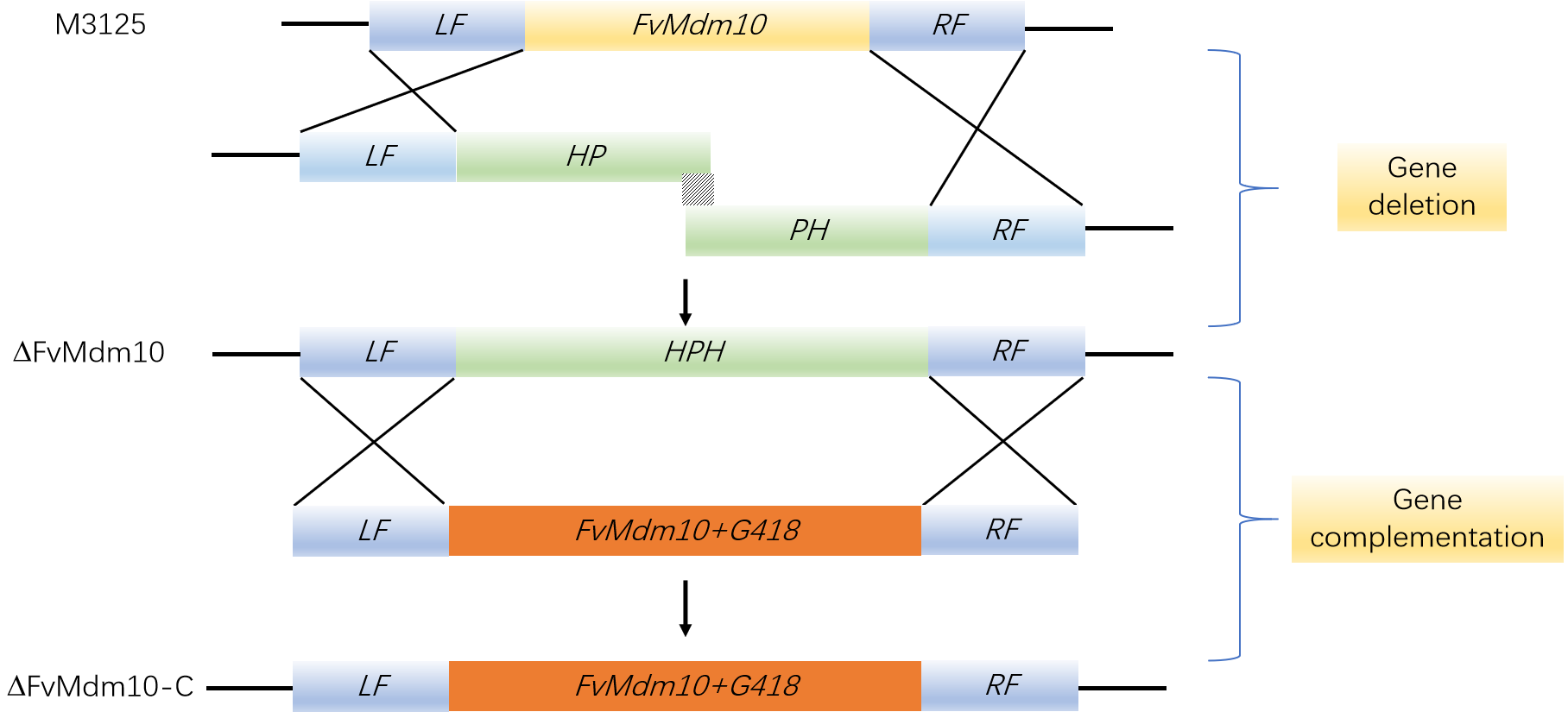

Supplement: Figure_S1.tif [file KVIR_A_2555419_SM8532.tif]

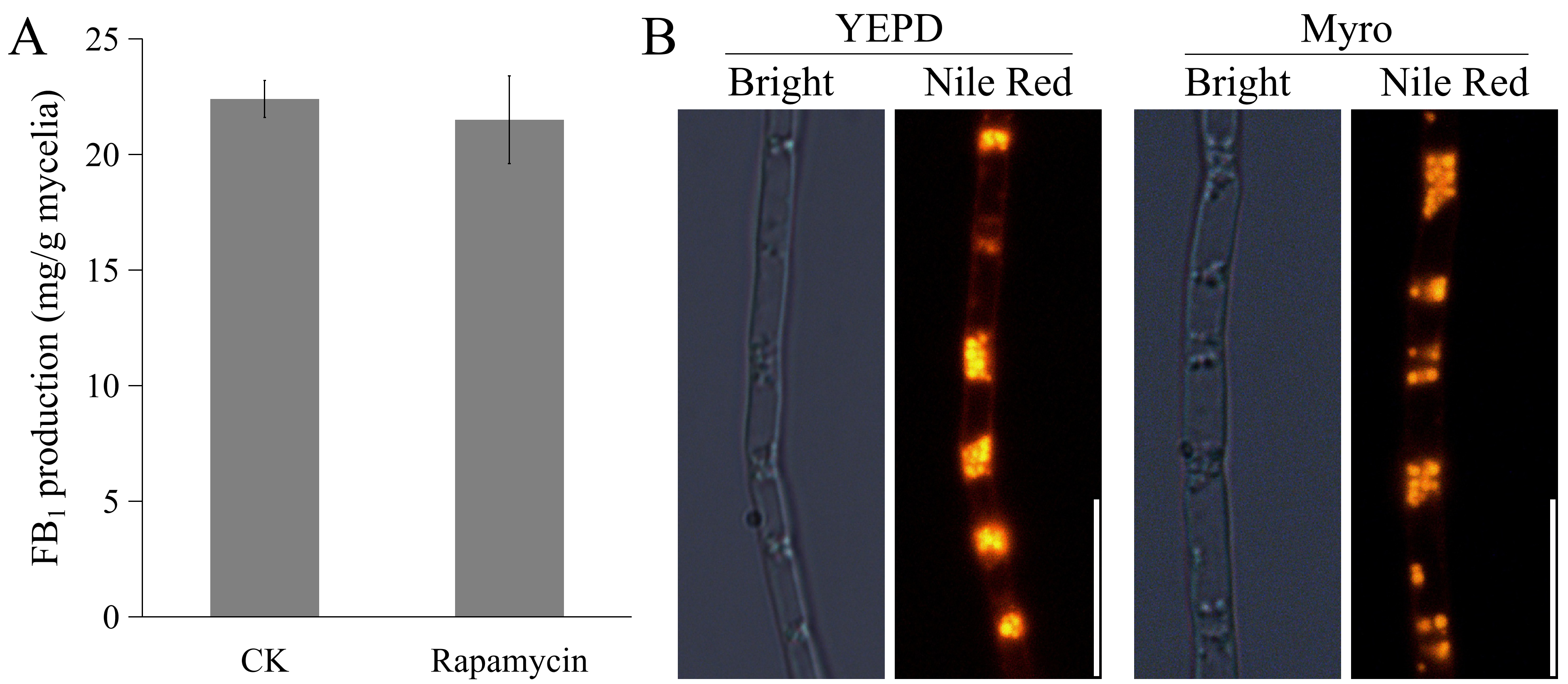

Supplement: Figure_S4.tif [file KVIR_A_2555419_SM8531.tif]
