# Supplementary material for: Functional role of FvMdm10 in stress response, pathogenicity, and fumonisins production in fusarium verticillioides
Source: Virulence. 2025 Sep 11;16(1):2555419. doi: 10.1080/21505594.2025.2555419 (PMC12427440; doi:10.1080/21505594.2025.2555419)
Supplement: Table S1.doc [file KVIR_A_2555419_SM8533.doc]

Table S1. **Primers used in this study.**

| Primer | Sequence (5’-3’) | Application |
| --- | --- | --- |
| Fum1_qF | GCTCTAGAGAACCGCACTATTC | For qPCR assay |
| Fum1_qR | TCAACTGGTACCGCCATATTC |
| Fum6_qF | GACACAGAGACTGGGCAAATA | For qPCR assay |
| Fum6_qR | GAAACTCCCATGAGGCTAACA |
| Fum8_qF | ATCACCGCCACTGTCTTTAC | For qPCR assay |
| Fum8_qR | GAAGCGTCGGACTTGATACTT |
| Fum21_qF | GGAAAGCTAACGACGGAGATAC | For qPCR assay |
| Fum21_qR | GACTCTTCCGCTGATCCATTAC |
| Vps13_qF | GCTCACGCTACTGGACTTTATC | For qPCR assay |
| Vps13_qR | CTCCGAATCGTCATCATCATCC |
| GAPDH_qF | CATCATCTCCAACGCCTCTT | For qPCR assay |
| GAPDH_qR | GTCATGAGACCCTCAACGATAC |
| Mdm10_qF | ACTTGGCTCAGTCGGTATTG | For qPCR assay |
| Mdm10_qR | GTAGATTGACCGTCTCGGATTG |
| Mdm10_LF/F | CATTGCTACTGTTGGTTTCC | Amplify *FvMdm10* 5’ flank sequence, for gene knock out |
| Mdm10_LF/R | ACCAGCCAGCCAACAGCTCCCCCTAGTGTGATGACTTGCCA |
| Mdm10_RF/F | AATACGCAAACCGCCTCTCCCCCATAGGTCCTGTCATCTACT | Amplify *FvMdm10* 3’ flank sequence, for gene knock out |
| Mdm10_RF/R | ACATTCTGTCTTCCGTCTGG |
| Mdm10_IN/F | CAACTTTGCGACATCCTACC | Amplify *FvMdm10* gene, for transformants screen |
| Mdm10_IN/R | GTTTAGGGTGCCGTAGTAGA |
| Mdm10_LF/F1 | GTTCAAAGCAAGCGGAGCA | Transformants screen, for gene knock out |
| Mdm10_RF/R1 | CCCCTGGATGAACTCTAACA |
| HPH-F | TTGGCTGGAGCTAGTGGAGGTCAA | Amplify *HY* fragment |
| HP-R | GTATTGACCGATTCCTTGCGGTCCGAA |
| PH-F | GTTCCCGGTCGGCATCTACTCTAT | Amplify *YG* fragment |
| HPH-R | GATGTAGGAGGGCGTGGATATGTCCT |
| Mdm10_R (GEN) | CAATATCATCTTCTGTCGACTCATGATGAAAACTGGATTTCC | For *FvMdm10* complementation |
| Mdm10_RF/F (GEN) | CTTGACGAGTTCTTCTGACATAGGTCCTGTCATCTACT |
| GEN_F | GTCGACAGAAGATGATATTG | Amplify *GEN* fragment |
| GEN_R | TCAGAAGAACTCGTCAAGA |
| FVEG_00326_qF | GTCCTTTGCCGTGAGGTATAG | For qPCR assay |
| FVEG_00326_qR | CAGTCGTCCACCTGTCATTT |
| FVEG_13610_qF | CCATACGGTCAATACGGTCAAC | For qPCR assay |
| FVEG_13610_qR | CCGTCTGCTTCTTCTGCATATC |
| FVEG_09866_qF | CATGGATTAGGAACCTGGAGAAG | For qPCR assay |
| FVEG_09866_qR | AGAAGAGATGCGCCAAGAAG |
| FVEG_04242_qF | CAGCTACGACACCTACAAGAAC | For qPCR assay |
| FVEG_04242_qR | GGGTCCAGCAAGCAAAGATA |
| FVEG_04241_qF | CCAGAAGAAAGCCTACCGATAC | For qPCR assay |
| FVEG_04241_qR | GGCATCAGGGTTGAGAAGAA |
| FVEG_04396_qF | CAGAGCATTGACTACGAGAGTG | For qPCR assay |
| FVEG_04396_qR | GGTGATCATGAGAGGAGTGTTC |
| FVEG_04346_qF | GCTGCTGACACGTTCAATTAC | For qPCR assay |
| FVEG_04346_qR | AAGGCTGTCGAAGGGTAAAG |
